# Inebriate Homes

**Published:** 1903-01-03

**Authors:** 


### Inebriate Homes.

The recently issued report of Dr. Welsh Branthwaite, Inspector under the Inebriates Acts, enters into many questions concerning the retreats established under these Acts, showing how much is being done to give the inebriate another chance in life, and also, unfortunately, how much remains to be done for the reclamation of this very hopeless class of cases. He reports that at the close of the year 1901, out of twenty-one institutions which had been licensed, more than half were being conducted by societies having philanthropic rather than commercial aims; a matter of some importance in view of the fact that a very large proportion of those who have given way to inebriety are—either as a cause or a consequence—so poor that they are able to pay little or nothing for their treatment or even for their support.

The report gives strong confirmation of the opinion, which is held by so many who have studied the subject, as to the inutility of detention for short periods, if anything like a cure is to be aimed at. The enforcement of long-continued abstinence from alcohol in every shape and form is essential if real good is to be done, and so fully is this recognised that the managers of some of the licensed retreats refuse to receive patients unless they will consent to be detained for a term extending for twelve months or more. There are many patients who, although ready enough to enter a retreat while suffering from the after effects of alcohol, are so full of self-confidence, perhaps tinged sometimes with a secret hope of before long tasting again of the forbidden pleasure, that as soon as they recover from their depression they refuse more than a temporary, and so far as cure is concerned quite inefficient, term of detention. Too often, all the care and money lavished upon the treatment of such cases is thrown away. They are a curse to their relatives, and the despair of the philanthropist. This brings us to the consideration of the recommendations made by Dr. Branthwaite in regard to the provision which is so urgently wanted for poor and semi-destitute cases.

Drink, in almost every station of life, causes monetary loss, but where it affects the breadwinner it quickly leads to such destitution that, except by aid of charity, effective treatment becomes impossible. All that can happen to such a man is to sink; to get such irregular employment as alone is left to irregular workers; to drop into the residuum, dragging down his family with him, and ultimately to become a permanent charge upon the rates. Clearly there is a field here for philanthropic effort. Yet it would be well, even in regard to this, that charitable people should take warning from the "ins and outs," to use a Poor Law term, for if charity is to step into the gap in dealing with the semi-destitute inebriates, it must be firmly laid down that it does so with the object of curing the patient of the vice, not merely of relieving him or her of the evil consequences it has entailed; and for this purpose charity must accept the guidance of experience which goes to show that a long term of compulsory detention is an essential part of the scheme of cure. The temporary reception of inebriates on an eleemosynary basis and their too early discharge on a "ticket of leave" would be to encourage rather than to check the prevalence of inebriety.

For mere convenience, however, and putting aside all question of cure, any community in which drunkenness prevails might well consider whether it might not be worth while to provide some sort of refuge to which the drunkard might be removed when he becomes outrageous in his behaviour. At the present time such patients—if patients we may call them—are a nuisance to all concerned. They are often sufficiently good workmen or even business men to earn a living perhaps even better than that enjoyed by many a steady man. But when they "break out" they become a curse to their homes, an anxiety to their friends, and a disgrace to their relatives, and unfortunately there is nowhere to put them. When they happen, under stress of circumstances, to be admitted to the wards of a hospital, they do a grievous injury to the other patients; they cannot be induced to "sign" for detention in a "home" for long periods, nor are the managers of inebriate homes in which the majority of the patients are long-time cases, generally willing to take them in, and they cannot as a rule be received in a lunatic asylum. There can be little doubt that, as Dr. Branthwaite suggests, there is room for institutions in which patients might be received who, although able to sign for short periods, would utterly refuse to submit to prolonged detention. These short-period retreats, although hardly aiming at cure, would be an immense convenience to friends and relatives, and in a certain but probably small percentage of cases they would give to the incipient drunkard—especially to such cases as have taken to drink under the stress of some temporary trouble—that which one would always be glad for even a criminal to receive, namely, another chance.

